# C-Reactive Protein in Atherothrombosis and Angiogenesis

**DOI:** 10.3389/fimmu.2018.00430

**Published:** 2018-03-02

**Authors:** Lina Badimon, Esther Peña, Gemma Arderiu, Teresa Padró, Mark Slevin, Gemma Vilahur, Gemma Chiva-Blanch

**Affiliations:** ^1^Cardiovascular Science Institute – ICCC, IIB-Sant Pau, Hospital de Sant Pau, Barcelona, Spain; ^2^CiberCV, Institute Carlos III, Madrid, Spain; ^3^School of Healthcare Science, Manchester Metropolitan University, Manchester, United Kingdom

**Keywords:** c-reactive protein, pentameric C-reactive protein, monomeric C-reactive protein, atherosclerosis, thrombosis, angiogenesis, ischemic heart disease, cardiovascular disease

## Abstract

C-reactive protein (CRP) is a short pentraxin mainly found as a pentamer in the circulation, or as non-soluble monomers CRP (mCRP) in tissues, exerting different functions. This review is focused on discussing the role of CRP in cardiovascular disease, including recent advances on the implication of CRP and its forms specifically on the pathogenesis of atherothrombosis and angiogenesis. Besides its role in the humoral innate immune response, CRP contributes to cardiovascular disease progression by recognizing and binding multiple intrinsic ligands. mCRP is not present in the healthy vessel wall but it becomes detectable in the early stages of atherogenesis and accumulates during the progression of atherosclerosis. CRP inhibits endothelial nitric oxide production and contributes to plaque instability by increasing endothelial cell adhesion molecules expression, by promoting monocyte recruitment into the atheromatous plaque and by enzymatically binding to modified low-density lipoprotein. CRP also contributes to thrombosis, but depending on its form it elicits different actions. Pentameric CRP has no involvement in thrombogenesis, whereas mCRP induces platelet activation and thrombus growth. In addition, mCRP has apparently contradictory pro-angiogenic and anti-angiogenic effects determining tissue remodeling in the atherosclerotic plaque and in infarcted tissues. Overall, CRP contributes to cardiovascular disease by several mechanisms that deserve an in-depth analysis.

## Introduction

C-reactive protein (CRP) is a short pentraxin belonging to the highly conserved family of calcium-dependent ligand-binding plasma proteins of the superfamily of soluble pattern-recognition molecules, and it is mainly found as a pentamer in the circulation. It is synthesized in the liver induced by interleukin (IL)-6 (1), IL-1β, and tumor necrosis factor (TNF) (2), although other tissues such as adipose tissue may be able to synthesize CRP under pro-inflammatory stimuli (3). The native circulating form of CRP is pentameric (pCRP), that is a disc of five identical subunits non-covalently bounded around a central pore (4). When pCRP bounds to one of its ligands [for instance lysophosphatidylcholine *via* activation of phospholipase A2 (5) or in denaturizing or oxidative environment (6)] it dissociates in a non-reversible manner into its non-soluble monomers, leading to a potential functional activation (7). pCRP and monomeric CRP (mCRP) are shown to exhibit different functions, although the specific physiopathological functions of CRP are still unknown and are a focus of intense research. It is believed that mCRP is involved in the innate immune system by activating the complement cascade (8), in angiogenesis (9) and in thrombosis (10), whereas pCRP is mostly released to the circulation after an inflammatory stimuli (1).

CVD is mainly caused by atherosclerosis, which starts from lipid infiltration in the vessel wall, endothelial dysfunction, and chronic low-grade inflammation causing plaque development that ends with clinical ischemic complications. Levels of pCRP in serum ≥3 µg/mL are used in the clinical setting as unspecific marker for inflammation, infection, and tissue injury, associated with an acute-phase response (11). Indeed, CRP is considered a predictor of future cardiovascular events (12), and in current guidelines is classified as Class III B level of evidence (13), although there are some discrepancies (14). CRP is a downstream biomarker of elevated IL-1, IL-6, and TNF-α. It can increase 10,000-fold within 6 h and has a half-life of 19 h, and its catabolic rate is independent of its plasma concentration (15). Besides its role in humoral innate immune response, CRP recognizes and binds multiple intrinsic ligands, such as the complement system, resulting in a significant increase in infarct size, cell receptors, apoptotic cells, growth factors, and extracellular matrix components, and thus contributing to cardiovascular disease progression. On those grounds, we aimed to highlight the implication of CRP and its forms on the pathogenesis of atherothrombosis and angiogenesis.

## CRP in Atherothrombosis

Atherothrombosis is a complex inflammatory pathological process initiated by lipid deposition in the arterial wall with a subse-quent recruitment of circulating leukocytes. The growing atheromatous plaque may become unstable and rupture, triggering the formation of a thrombus by accumulation of platelets and coagulation proteins. Occlusive thrombi may eventually induce an ischemic event (16). In this process, inflammation has a pivotal role in all phases, and CRP actively participates by activating the complement system, and inducing apoptosis, vascular cell activation, leukocyte recruitment, lipid accumulation, platelet aggregation, and finally thrombosis (17). mCRP is detectable in the vessel wall in early stages of atherogenesis but not in healthy vessels, and accumulates during the progression of atherosclerosis, whereas pCRP is not detectable in healthy or atheroscle-rotic vessels (18). In this context, complement activation by enzymatically modified low-density lipoprotein (LDL) plays an important role in atherogenesis (19). Enzymatic modification of LDL confers the capacity to bind pCRP, and CRP-binding enhances complement activation through C3 cleavage (20). Both pCRP and mCRP are able to activate and amplify the classical pathway of the complement system by interacting with the complement factor C1q, significantly activating C1. Only mCRP is able to interact with complement factor H and C4b-binding protein (21), thus provoking local inflammatory responses and contributing to the establishment and progression of atherosclerosis or to the tissue damage following myocardial infarction. In addition, pCRP can promote inflammation by binding to modified or oxidized LDL and (non) oxidized phosphatidylcholine from apoptotic cells (22), promoting the transformation from macrophages to foam cells.

C-reactive protein contributes to endothelial dysfunction and hypertension by inhibiting nitric oxide (23), increasing endothelin-1 production, and thus impairing endothelial-dependent vascular relaxation (24). In the setting of chronic local inflammation in atherosclerosis, the addition of mCRP to apical but not basolateral surfaces of intact human coronary artery endothelial cell monolayers, upregulated monocyte chemotactic protein (MCP)-1, IL-8, and IL-6 expression and activated endothelial cells through the polarized induction of phospholipase C, p38 mitogen-activated protein kinase, and nuclear factor (NF)-κB signaling pathways (25). Therefore, tissue-associated mCRP induces endothelial cell activation and dysfunction, and spatial localization is determinant for the highly context-dependent actions of CRP isoforms within vessels.

In addition, pCRP contributes to plaque instability by activating NF-κB and, therefore, increasing endothelial cell adhesion molecules expression such as vascular cellular adhesion molecule-1, vascular E-selectin, and MCP-1 (26, 27). pCRP also induces monocyte polarization to M1 and conversion from M2 to M1 phenotype (2), thus promoting monocyte recruitment into the plaque. Indeed, circulating pCRP binds to the cell membrane of activated, but not resting monocytes (28), and activated but not resting platelets and apoptotic leukocytes are able to dissociate pCRP to mCRP *via* lysophosphatidylcholine inducing reactive oxygen species (ROS) production and monocyte chemotaxis, activation and adhesion (18), being mCRP and not pCRP the responsible of these effects, even at low concentrations. In neutrophils, mCRP but not pCRP increases IL-8, CD11b/CD18, and superoxide production, and induces endothelial nitric oxide synthase-mediated nitric oxide formation. This leads to enhanced peroxynitrite formation, and to the activation of NF-κB and activator protein (AP)-1 (29), as well as enhanced neutrophil adhesion to activated endothelial cells (30), thus aggravating the inflammatory response at injured vascular sites and contributing to plaque destabilization. Overall, mCRP is able to aggravate the preexisting inflammatory response by inducing leukocyte rolling, adhesion, and transmigration to the endothelium and generation of ROS (5), which in turn, modifies the structure and ligand recognition function of CRP (31).

In addition, CRP also contributes to plaque instability by inducing the expression of metalloproteinases (MMP) 1, 2, and 9 (32, 33). On those grounds, CRP mRNA was detected in potentially vulnerable ulcerated carotid artery plaques but not in hemorrhagic ulcerated plaques independently of the circulating levels of CRP. In non-complicated ulcerated carotid artery plaques, CRP was mainly localized in infiltrated and endothelial cells around areas of newly formed microvessels (34), potentially contributing to plaque neovascularization and rupture resulting in thrombosis.

C-reactive protein also contributes to thrombosis, but depending on its form it elicits different actions. CRP at 10–100 mg/L is able to increase 75-fold tissue factor (TF) procoagulant activity of monocytes, with a parallel increase in TF antigen levels (35). CRP at 2–24 mg/L activates both inflammation and coagulation through increasing circulating levels of E-selectin, von Willebrand factor, IL-6, IL-8, serum amyloid A protein, type II secretory phospholipase A_2_, prothrombin F1 +2, D-dimer, and plasminogen activator inhibitor type-1 (36). It has been shown that circulating microvesicles can bind pCRP and dissociate it to mCRP, and patients with myocardial infarction have circulating microvesicles carrying mCRP (37). In addition, pCRP binding to activated cell-derived microvesicles also undergoes a structural change leading to the expression of neoepitopes without disrupting the pentameric symmetry activating the classical complement pathway through C1q binding and enhancing leukocyte recruitment to inflamed tissues (28). pCRP has no involvement in thrombogenesis, whereas mCRP is able to promote thrombosis by inducing platelet activation (38), platelet adhesion by upregulating P-selectin (10), and thrombus growth (39). Additionally, mCRP has been found in platelet aggregates and stimulates further platelet deposition (38). Blocking glycoprotein IIb-IIIa on activated platelets prevented the dissocia-tion of pCRP to mCRP and reduced platelet deposition at the arterial wall (38).

**Figure 1:**
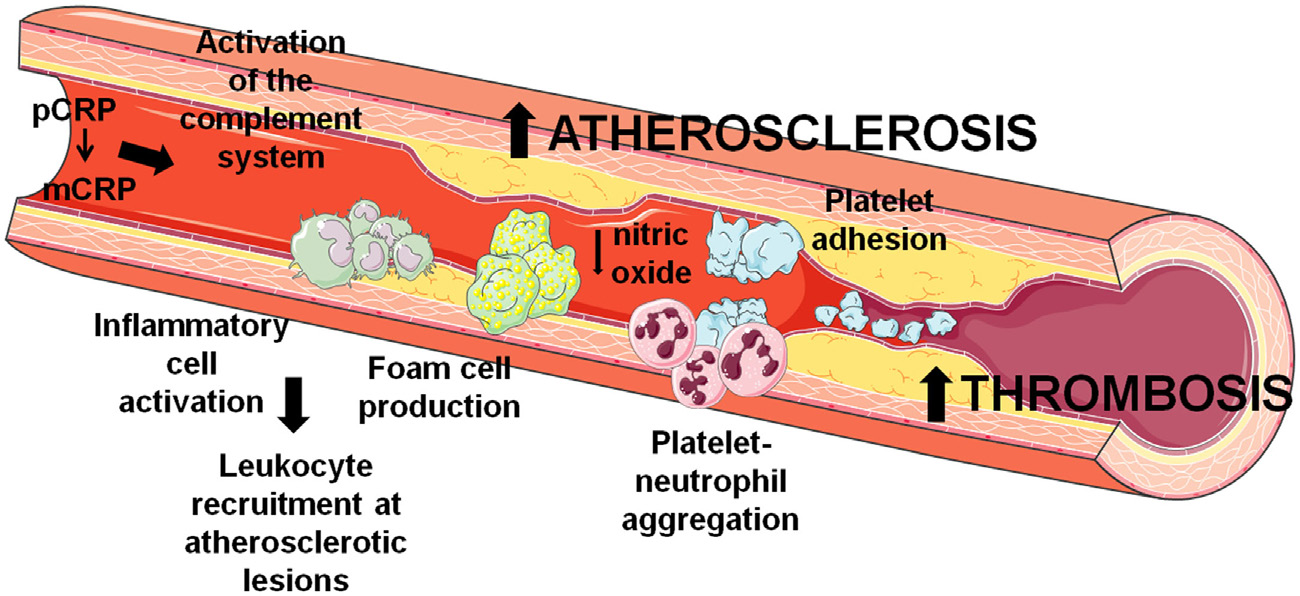
Involvement of C-reactive protein (CRP) in atherothrombosis. CRP contributes to the development and progression of atherosclerosis and thrombosis by several mechanisms that induce endothelial dysfunction, leukocyte recruitment at atherosclerotic lesions, and thrombus formation through platelet activation and aggregation.

As depicted in Figure 1, the dissociation of pCRP into mCRP could be interpreted as a master switch for the inflammatory processes involved in atherogenesis. Both mCRP bound to phosphorylcholine of activated cell membranes and mCRP present within the advanced atherosclerotic plaque may play a critical role in the further development of the plaque and thrombus formation and propagation upon mechanical or spontaneous atherosclerotic plaque rupture. Nevertheless, it is worth mention that some *ex vivo* experiments suggest anti-atherosclerotic functions of CRP. As previously stated, CRP binds to enzymatically modified LDL at the same binding site as phosphocholine; therefore, it could prevent the formation of foam cells and limit complement activation (40, 41). Indeed, when CRP binds to lysophosphatidylcholine, this complex triggers a less potent generation of ROS and less activation of the transcription factors AP-1 and NF-κB by macrophages in comparison to free CRP or lysophosphatidylcholine, reducing the pro-atherogenic effects of macrophages (42). Finally, it has also been shown that CRP also affects the physicochemical properties of LDL and inhibits further oxidation of ox-LDL (43, 44), although the mechanism remains unknown. In addition, mCRP has been found to decrease the uptake of acetylated LDL by endothelial cells independently of CD16, CD32, or the receptor for oxidized LDL (45).

## CRP in Ischemia and Angiogenesis

Monomeric CRP has been involved in ischemic heart disease (46), therefore pCRP dissociation to mCRP modulates inflammation in both acute (cardiac ischemia/reperfusion) and chronic (atherosclerosis) inflammatory processes. Local inflammatory response during myocardial ischemia contributes to myocardial damage and infarct size, and plays a major role in angiogenesis and tissue remodeling. Infiltrated macrophages at the border site of the cardiac ischemic lesion express mCRP (47, 48), and CRP in monocytes upregulates vascular endothelial growth factor (VEGF)-A expression *in vitro via* binding to its Fc-gamma receptors (49). In fact, myocardial ischemia activates mCRP expression in myocardial infiltrated macrophages but not in peripheral blood mononuclear cells (47), and cardiac mCRP expression re-mains elevated after 1 week of acute myocardial infarction (48), potentially contributing to cardiac remodeling and in perpetuating and/or amplifying the inflammatory process. Along this line, circulating CRP has been shown to correlate with infarct size and left ventricle remodeling 2 months after percutaneous coronary intervention, and patients with persistent microvascular obstruction presented increased circulating CRP levels 2 days after percutaneous coronary intervention (50).

Angiogenesis also has a role in plaque instability and disruption, favoring leukocyte and macrophage infiltration in growing atherosclerotic lesions. Indeed, the adventitial *vasa vasorum* facilitates neovascularization related to progression of atherosclerosis (51). Although some authors have observed that CRP inhibits VEGF production and angiogenesis (23, 52), several studies suggest that mCRP may be a mediator of neovessel formation in the intima of vulnerable plaques, as it has been localized in the adventitia and intimal neovessels from complicated regions of unstable carotid plaques (53). In the setting of atherosclerosis, CRP upregulates VEGF expression *via* activating hypoxia inducible factor-1α, and MMP-2 expression and in adipose-derived stem cells, significantly increasing endothelial cell tube formation and *vasa vasorum* proliferation (54). As previously explained, mCRP has been localized around newly formed microvessels in carotid artery plaques and in peri-infarct regions after an acute ischemic stroke (34, 55), promoting angiogenesis and inducing inflammation and increased permeability of abnormally developing microvessels after tissue injury (56), potentially leading to an increased risk of dementia.

**Figure 2:**
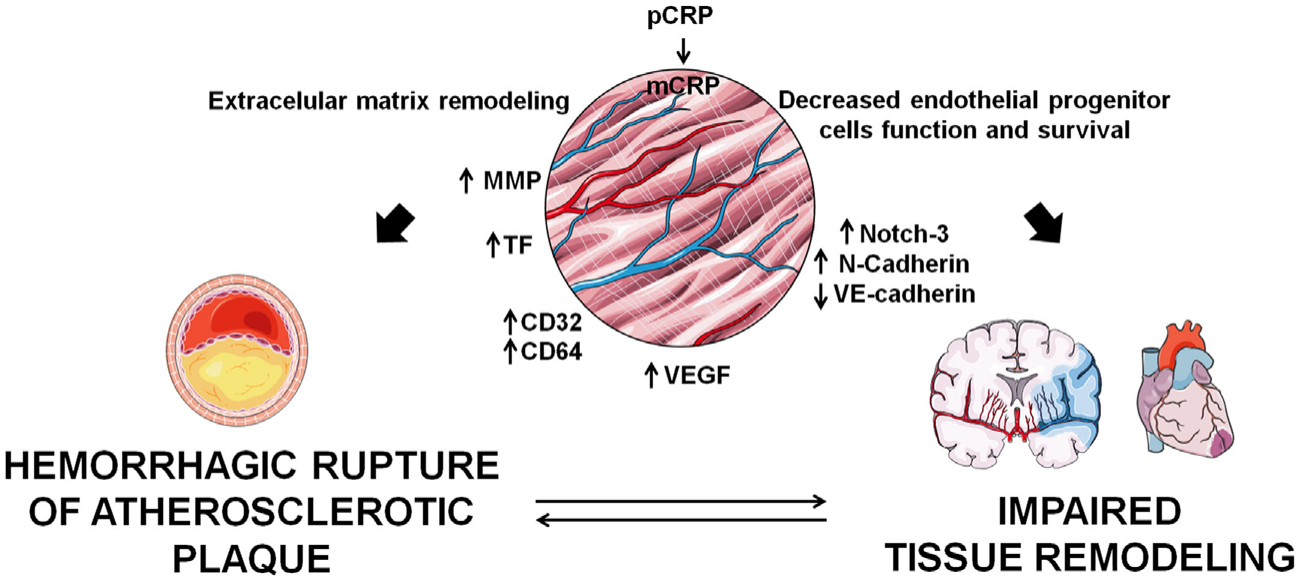
Implications of C-reactive protein (CRP) in angiogenesis. monomeric CRP (mCRP) has apparently contradictory pro-angiogenic and anti-angiogenic effects which determine tissue remodeling in the atherosclerotic plaque and in infarcted tissues.

In stroke patients, mCRP colocalized with endoglin (CD105), a marker of angiogenesis in regions of revascularization, and stimulated phosphorylation of extracellular signal-regulated kinase (ERK)1/2, inducing cell migration and formation of tube-like structures independently of the CD16 axis (55). mCRP exerts potent angiogenic effects on microvascular endothelial cells. CRP dissociates into mCRP on the endothelial cell membrane and mCRP induces angiogenic effects by increasing TF expression and activation of the axis F3-TF-ETS1-CCL2 (9), and by increasing endothelial expression of CD32 and CD64 (57), thus promoting migration, wound repair, and tube-like formation. In parallel, it has been demonstrated that mCRP has the ability to promote angiogenesis by increasing proliferation, migration, and tube-like structure formation *in vitro* and by stimulating blood vessel formation *in vivo* with the chorioallantoic membrane assay. mCRP induced vascular VEGFR2/KDR, platelet-derived growth factor (PDGF-BB), inhibitor of DNA binding/differentiation-1 (ID1) gene expression, notch family transcription factors (Notch1 and Notch3), and also induced stabilization and maturation of cysteine-rich angiogenic inducer 61 (CYR61/CCN1), overall playing a central role in the main stages of blood vessel formation and remodeling (58). Along this line, mCRP induces Notch-3 and N-cadherin expression and downregulates VE-cadherin expression. mCRP and Notch-3 act in a co-operative manner in vascular endothelial cells, exerting a role in the remodeling and maturation of the vascular development by increasing endothelial cell proliferation, migration, and tube formation and also stabilizing vascular structures through modulating VE-cadherin and N-cadherin expression (59). On the other hand, CRP has several deleterious effects on endothelial progenitor cells, which account for about 26% of endothelial cells in newly formed blood vessels (60), by decreasing their survival and inducing apoptosis, by impairing their differentiation through the inhibition of the expression of tyrosine-protein kinase receptor for angiopoietin (Tie)-2, endothelial cell-specific lectin, and VE-cadherin, and by impairing nitric oxide-dependant angiogenesis *via* decreasing endothelial nitric oxide synthase (61, 62). Therefore, as depicted in Figure 2, angiogenesis plays a dual role in CVD progression, and further research should focus on the mechanisms by which CRP may contribute to the atherosclerotic process and/or tissue repair.

## CRP and CVD Prognosis

Although CRP response is unspecific and is triggered by many disorders unrelated to cardiovascular disease, mathematical models that incorporate high-sensitivity CRP (hsCRP) improve CV risk prediction. Increased levels of CRP strongly predict the thrombotic complications of atherosclerosis, principally myocardial infarction (1) and its adverse outcomes such as left ventri-cular failure, cardiac death, and ventricle rupture (7). In fact, CRP may have a role in risk stratification of patients with established CVD. hsCRP levels > 3 mg/L are predictive of major adverse cardiac events at 1 year, and are also associated with higher coronary plaque burden and volume (63). In addition, low, average and high CV risk categories can be stratified by hsCRP levels (<1.0, 1.0 to 3.0, and >3.0 mg/L, respectively) (64), and in the general population CRP levels are able to independently predict the risk of all-cause and cardiovascular mortality (65).

In subjects at intermediate risk of CVD, incorporation of CRP to a model of assessment of CV risk improves the prognostic power for myocardial infarction presentation (66), and could help prevent 1 additional CV event in 10 years from 400–500 screened subjects (12). In patients with previous CVD and in asymptomatic subjects, hsCRP was a moderated predictor of coronary heart disease at the long term (67). In fact, the combination of troponin I, N-terminal pro-brain natriuretic peptide (NT pro-BNP), cystatin C and CRP improved significantly the risk stratification for cardiovascular death (68). In patients with stable and unstable angina, elevated CRP levels are predictive of future coronary events (69). Indeed, in ST-elevation myocardial infarction patients CRP levels predicted heart failure and cardiovascular mortality the year after the CV event (70), and in patients with non-ST-elevation myocardial infarction, in-hospital mortality was four times higher in patients with a CRP > 10 mg/L compared to patients with <3 mg/L CRP levels, and this association persisted at the long term (71).

High-sensitivity CRP can be quantified by immunonephelometry sensitized techniques routinely used to measure circulating pCRP with a lower detection limit than former procedures. However, as stated in this review it is unlikely that circulating pCRP elicit a direct role in CHD progression (7), because no major prothrombotic or pro-inflammatory effects have been found for circulating pCRP, and no association between genetically elevated CRP and risk of CHD has been found (72). Therefore, it seems plausible that mCRP would be the responsible for the observed associations between CRP and CVD.

## Conclusion

As reviewed, mCRP is a potential regulator of signaling pathways associated with thrombosis, angiogenesis, and inflammation. The ability of CRP to bind and interact with multiple ligands underscores its implication in different steps of atherosclerosis and CVD. CRP contributes to atherosclerosis progression by exerting pro-inflammatory effects, modulating the innate immune response and activating the complement system, promoting platelet activation, thrombus formation, vascular remodeling, and angiogenesis. However, whether CRP acts as regulator or amplifier of the innate immune response remains to be fully elucidated. Determining whether increased pCRP production merely reflects atherosclerosis or does indeed participate in its pathogenesis and complications is of utmost importance in order to definitively consider hsCRP as a clinical biomarker of CVD.

The study of the molecular mechanisms by which CRP con-tributes to atherothrombosis, angiogenesis, and CVD has a major pitfall. Human CRP does not interact with C1q in mice, and mice do not produce large amounts of CRP after an inflammatory stimuli (46). The study of CRP function has largely been performed with administration of exogenous, heterologous CRP or with mice transgenic for rabbit or human CRP. Therefore, caution should be taken when extrapolating from animal models to humans, and more research toward a more appropriate animal model is still warranted. Taking this into consideration, further research is required in order to differentially characterize the roles of CRP isoforms (pCRP, facilitator, versus mCRP, effector) in CVD onset and progression, and the binding ligands to circulating pCRP which can lead to CRP dissociation and induction of local inflammation in order to develop more potent and orally bioavailable “CRP inhibitors” for the treatment of inflammation and atherosclerosis.

## Author Contributions

GC-B prepared the main body of the manuscript and figures. EP, GA, TP, MS, and GV wrote different sections of the manuscript. LB revised and prepared the manuscript. All authors listed critically revised the paper for intellectual content.

## Conflict of Interest Statement

The authors declare that the research was conducted in the absence of any commercial or financial relationships that could be construed as a potential conflict of interest.
